# How to transform a general hospital into an “infectious disease hospital” during the epidemic of COVID-19

**DOI:** 10.1186/s13054-020-02864-z

**Published:** 2020-04-14

**Authors:** Hongli He, Caiying Hu, Nian Xiong, Cheng Liu, Xiaobo Huang

**Affiliations:** 1 Department of Critical Care Medicine, Sichuan Provincial People’s Hospital, University of Electronic Science and Technology of China, No. 32 West Section 2, First Ring Road, Chengdu, Sichuan China; 2 Science and Education Department, Wuhan Red Cross Hospital, No. 392 Hongkong Road, Wuhan, Hubei China; 3 Department of Neurology, Union Hospital, Tongji Medical College, Huazhong University of Science and Technology, 1277 Jiefang Avenue, Wuhan, Hubei China; 4 Wuhan Red Cross Hospital, No. 392 Hongkong Road, Wuhan, Hubei China; 5 Medical Service Center of Sichuan Province, No.2-3 South Yulin Street, Chengdu, Sichuan China

Dear Editor,

The newly confirmed coronavirus disease 2019 (COVID-19) cases are still increasing strikingly in many countries according to the data reported by the World Health Organization. Liu et al. suggested that reconstructing an existing hospital into an infectious disease hospital (IDH) is an important strategy to prepare for the epidemic [1]. Here, we will extend their advice and share some of our lessons.

Wuhan Red Cross Hospital is a secondary general hospital with 500 beds. According to the instruction of the Chinese government, the hospital became a designated hospital which only received the febrile patients since January 22. The hospital responds to it very quickly. First, all the uninfected patients were transferred to other hospitals. Second, the sixteen-story hospital building was redesigned, and the changes are shown in Fig. 1a. Most importantly, two independent observation area and intensive care units (ICU) were rebuilt for COVID-19 test negative and positive patients; the ICU beds should occupy 26.1–32% of total beds [2, 3]. Third, twelve fever clinics were set to triage the patients; the protocol is shown in Fig. 1b. Fourth, infection prevention and control is very important: (a) training of the medical staff through face to face and video; (b) independent accesses for patients and medical staff; (c) the staff enter and exit the contaminated zone via two isolated aisles; (d) an infection control team checks each step of putting on and taking off personal protective equipment (PPE) to make sure that the staff is doing correctly; and (e) the doctors take 4–8 h shift, and the nurses take 4 h shift to reduce the exposure risk. Fifth, prepare enough PPEs, medical equipment like high-flow nasal cannula, ventilator, bronchoscope, sterilizing equipment, and extracorporeal membrane oxygenation (ECMO) if possible. Sixth, sufficient oxygen supply is very crucial for these patients as all the patients need oxygen therapy; unfortunately, some of our patients died because of the shortage of oxygen at the early time. Seventh, with the help of the government, several medical teams including doctors and nurses from infectious disease, pulmonary department, and ICU from other provinces came to help us. The specialists from the three departments were the leader of the new wards. Eighth, the treatment of the patients was according to the guidelines updated by the National Health Committee of China. A multidisciplinary team was formed to help to make a treatment plan for the critically ill patients, and sometimes, the teleconsultation was held for the patients either.

**Fig. 1:**
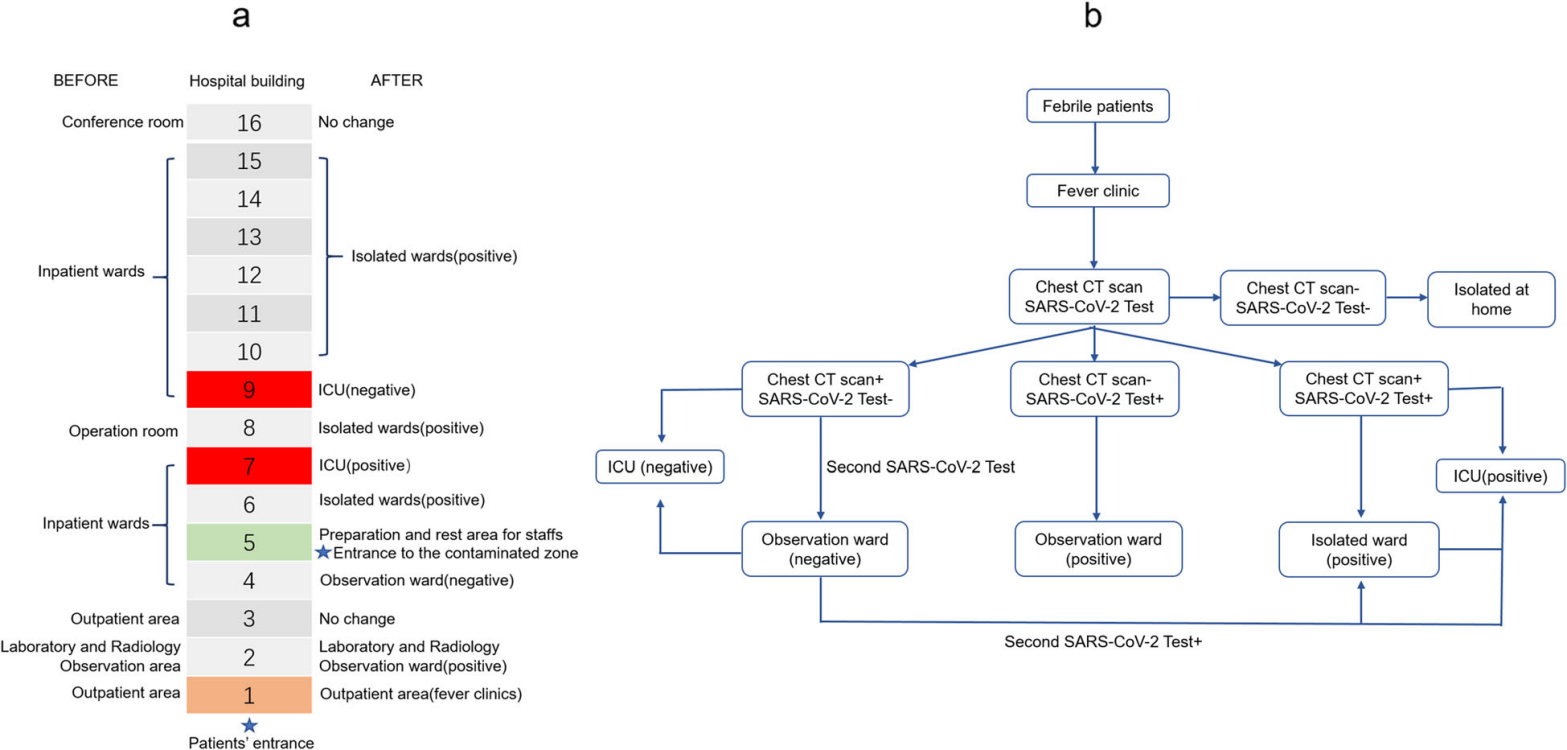
Schematic of the hospital modification (**a**) and the flow chart of the treatment process of suspected COVID-19 patients (**b**)

## Data Availability

Not applicable.

## Abbreviations

COVID-19: Coronavirus disease 2019
IDH: Infectious disease hospital
ICU: Intensive care unit
PPE: Personal protective equipment
ECMO: Extracorporeal membrane oxygenation
